# The feasibility and safety of complete laparoscopic extended right hemicolectomy with preservation of the ileocecal junction in right-transverse colon cancer

**DOI:** 10.1186/s12957-020-01922-8

**Published:** 2020-07-07

**Authors:** Hao Su, Hongliang Wu, Bing Mu, Mandula Bao, Shou Luo, Chuanduo Zhao, Qian Liu, Xishan Wang, Zhixiang Zhou, Haitao Zhou

**Affiliations:** 1 Department of Colorectal Surgery, National Cancer Center/National Clinical Research Center for Cancer/Cancer Hospital, Chinese Academy of Medical Science and Peking Union Medical College, No. 17, Pan Jia Yuan Nan Li, Chaoyang District, Beijing, 100021 People’s Republic of China; 2 Department of Anesthesiology, National Cancer Center/National Clinical Research Center for Cancer/Cancer Hospital, Chinese Academy of Medical Science and Peking Union Medical College, Beijing, 100021 China

**Keywords:** Ileocecal junction-preserved, Colon cancer, Complete laparoscopy, Right hemicolectomy, Bowel function

## Abstract

**Background:**

To evaluate the feasibility and safety of a new surgical method, complete laparoscopic extended right hemicolectomy with preservation of the ileocecal junction in right-transverse colon cancer.

**Methods:**

We retrospectively analyzed and compared the data of consecutive patients with right-transverse colon cancer who underwent complete laparoscopic extended right hemicolectomy with preservation of the ileocecal junction (*n* = 23) and conventional complete laparoscopic extended right hemicolectomy (*n* = 34) in our hospital between October 2017 to May 2019, respectively.

**Results:**

The overall operation time of the ileocecal junction-preserved group was significantly shorter than that of the control group (*p* = 0.048). There was no difference in the number of harvested lymph nodes, metastatic lymph nodes, and rate of metastatic lymph nodes (*p* > 0.05). The ileocecal junction-preserved group showed shorter time of first flatus, lower frequency of postoperative diarrhea, and shorter duration of postoperative hospitalization. Furthermore, it also showed that the defecation frequency was lower in the ileocecal junction-preserved group than the control group on the 1st, 3rd, and 6th month (*p* < 0.05), and the number of patients who defecated at night or defecated four times or more a day was less in the ileocecal junction-preserved group than control group on the 1st month (*p* < 0.05).

**Conclusion:**

The complete laparoscopic extended right hemicolectomy with preservation of the ileocecal junction promises as a safe and feasible surgical procedure for right-transverse colon cancer, associated with earlier recovery of bowel function, shorter operation time, and similar pathological outcomes when compared to the conventional laparoscopic procedure.

## Introduction

According to the International Agency for Research on Cancer (IARC), over 1.8 million new colorectal cancer cases and 881,000 deaths were estimated to occur in 2018, and colorectal cancer ranked third in terms of cancer incidence and second in terms of cancer mortality [1]. The surgical treatment for colorectal cancer has improved significantly with technical advances and theoretical progress [2], while the surgical procedures for transverse colon cancer can be controversial because of the location of the tumor [3, 4]. For right-transverse colon cancer, the conventional surgical procedure is laparoscopic extended hemicolectomy with extracorporeal anastomosis.

However, it was found that a conservative approach to transverse colon cancer is feasible and safe, and oncological outcomes are comparable between transverse colectomy and extended hemicolectomy in a systematic review and meta-analysis for patients with transverse colon cancer [5]. Some studies also recommend that a distance of 10 cm from the tumor is adequate for longitudinal resection margin in colon cancer because they found that the longitudinal spread of lymph nodes greater than 10 cm beyond the tumor is extremely rare [6, 7]. In fact, the surgeon is able to perform a less invasive surgery on the basis of radical oncological outcomes. Since the development of intracorporeal anastomosis [8], complete laparoscopic extended hemicolectomy with preservation of the ileocecal junction may be an optional procedure for right-transverse colon cancer. Therefore, we try to apply this procedure in the laparoscopic treatment for right-transverse colon cancer and explore its safety and feasibility. We assume that the preservation of ileocecal junction may be helpful for earlier recovery of bowel function. The short-term outcomes of this procedure compared with conventional complete laparoscopic extended hemicolectomy were presented in this study.

## Methods

### Patients

We retrospectively collected data for 70 patients with right-transverse colon cancer who underwent complete laparoscopic surgery in our hospital between October 2017 and May 2019. All the patients were diagnosed with colon adenocarcinoma by colonoscopy. The right-transverse colon is defined as the right one-third of the transverse colon in this study. Exclusion criteria for the study included multiple colorectal primary carcinomas, a history of past colonic surgery, stage IV colon cancer, and emergency surgery for bowel obstruction and/or perforation. A total of 57 patients were included in this study and grouped into the ileocecal junction-preserved group (*n* = 23) who underwent complete laparoscopic extended hemicolectomy with preservation of the ileocecal junction and the control group (*n* = 34) who underwent conventional complete laparoscopic extended hemicolectomy. The study was conducted in accordance with the principles of the Declaration of Helsinki and was approved by the ethics committee of Cancer Hospital, Chinese Academy of Medical Sciences. The procedure used during this study was explained to all the patients in detail prior to the surgery, and every patient provided a written informed consent for surgery.

Medical reports were reviewed to extract information regarding diverse clinical parameters, including age, gender, body mass index (BMI), American Society of Anesthesiologists (ASA) score, previous abdominal operation history, and preoperative chemotherapy. The surgical factors collected included operative time, estimated blood loss, and removal method of the specimen. Pathological outcomes included the length of the tumor, the proximal resection margin, the distal resection margin, the number of harvested lymph nodes, metastatic lymph nodes, rate of metastatic lymph nodes, and the pathological TNM stage. The time to ground activities, fluid diet intake, first flatus, and postoperative hospitalization were collected as factors associated with postoperative general recovery.

Postoperative diarrhea was defined as abnormal feces, such as loose stool, watery stool, mucous stool, or bloody purulent stool three times or more a day within 2 weeks after the operation. Other complications including anastomotic bleeding, anastomotic stenosis, anastomotic leakage, wound infection, urosepsis, pneumonia, lymphorrhea, abdominal infection, incisional hernia, and bowel obstruction were defined according to the Clavien–Dindo classification [9]. Mean defecation frequency, the number of patients who defecated at night (at least once a week), or defecated four times or more a day (at least once a week) were recorded on the 1st, 3rd, 6th, and 12th month after surgery to evaluate the postoperative recovery of bowel function.

### Surgical procedures

Under general anesthesia, all patients were placed in the supine lithotomy position, and a five-port technique was used. A D3 LN dissection was performed in all patients.

**Fig. 1:**
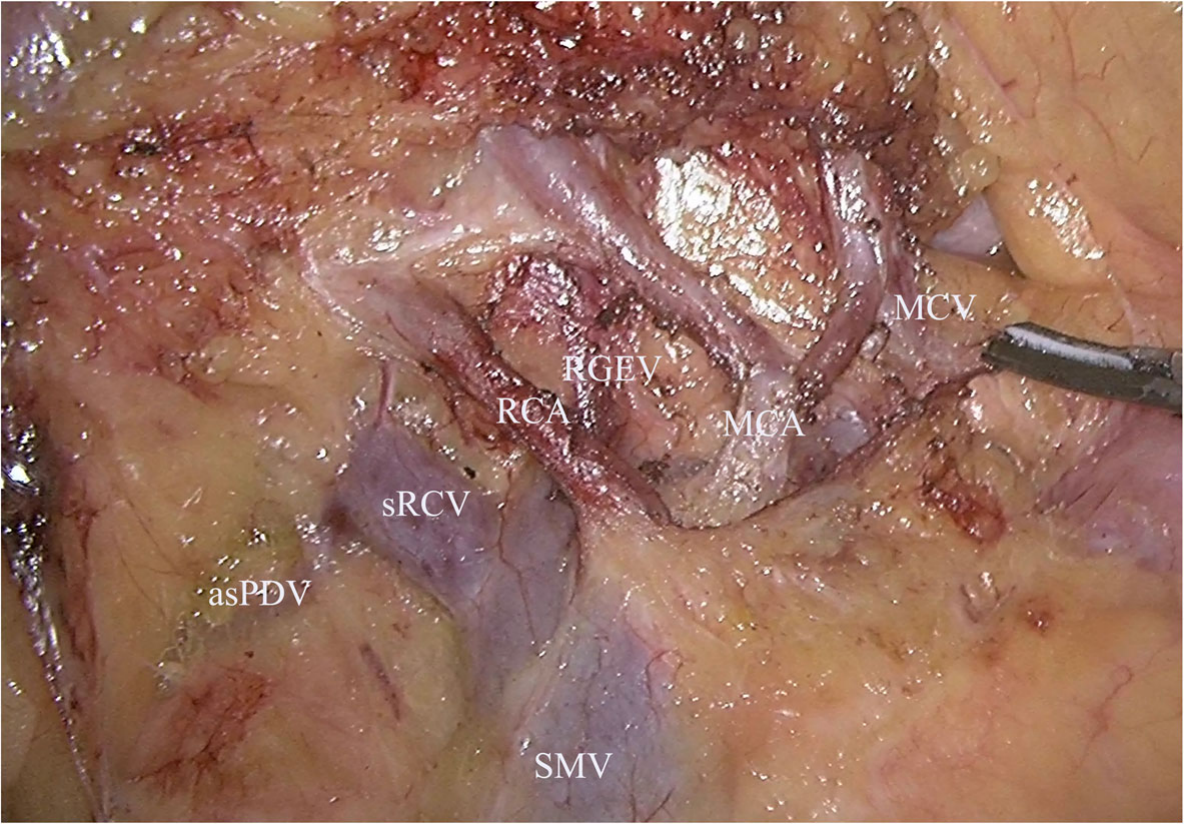
D3 lymphadenectomy in the complete laparoscopic extended right hemicolectomy with preservation of the ileocecal junction. SMV, superior mesenteric vein; RGEV, right gastroepiploic vein; sRCV, superior right colic vein; RCV, right colic artery; asPDV, anterior superior pancreaticoduodenal vein; MCV, middle colic vein; MCA, middle colic artery

**Fig. 2:**
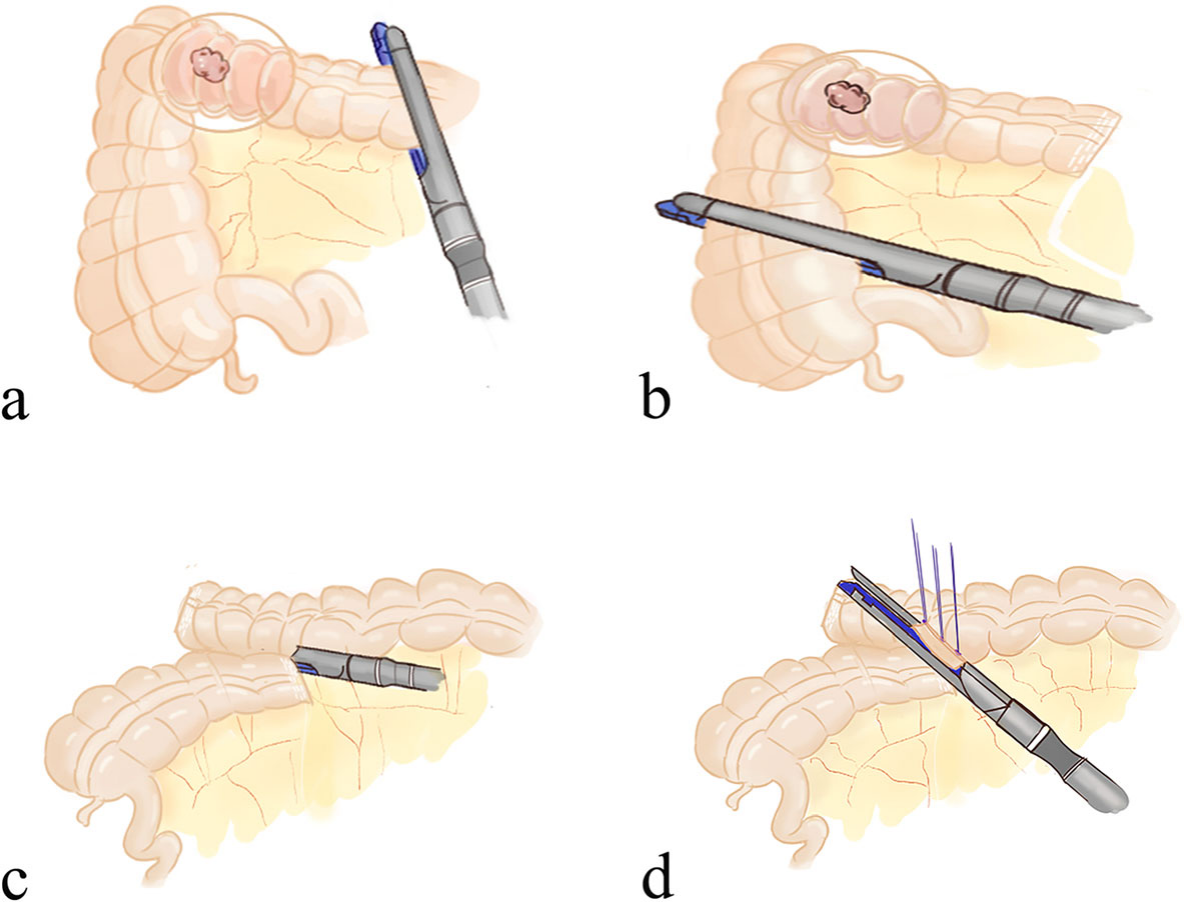
Surgical procedures of anastomosis in the complete laparoscopic extended right hemicolectomy with preservation of the ileocecal junction. **a** The disarticulation of the proximal intestines 10 cm from the tumor. **b** The disarticulation of the distal intestines 10 cm from the tumor. **c** The two intestinal walls were approximated and joined. **d** The enterotomy was closed with one linear stapler

**Fig. 3:**
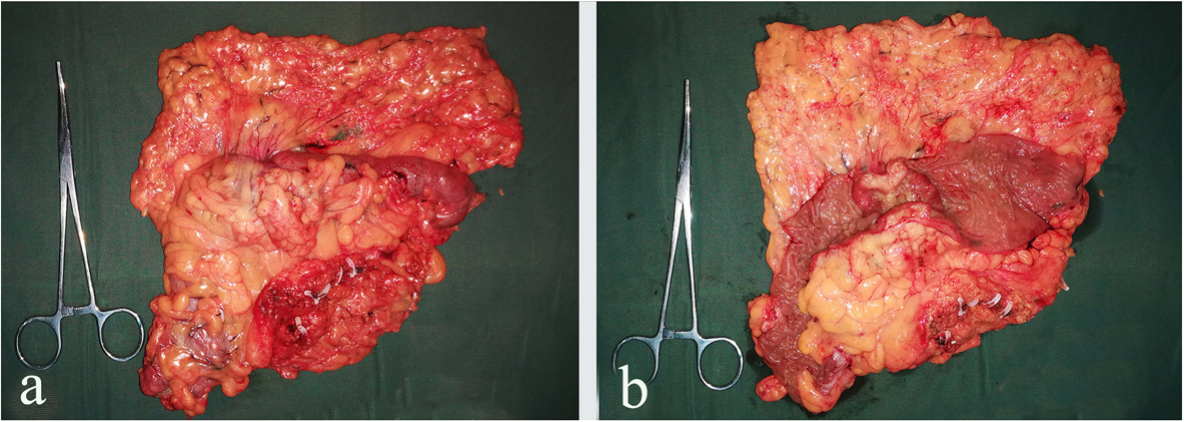
Specimen from the complete laparoscopic extended right hemicolectomy with preservation of the ileocecal junction. **a** Colonic serous membrane. **b** Colonic mucosal membrane

For the ileocecal junction-preserved group, a medial-to-lateral approach was used for the exposure of the mesentery with the assistance of the peritoneal fixation of the right colon laterally. The ileocecal junction was freed, and the ileocecal junction and the ileocolic vessels were preserved. Considering the inferior of the pancreatic neck as the anatomical landmark, the “mesenteric window” was opened. After skeletonizing the superior mesenteric vein (SMV) cranially, we divided and ligated the right colic vessels (if they existed), the superior right colic vein (sRCV), the right gastroepiploic vein (RGEV), the middle colic artery (MCA), and the middle colic vein (MCV) at their origins one by one (Fig. 1). A hypopyloric lymphadenectomy was performed along with the original ligation of the right gastroepiploic artery (RGEA). Relevant parts of the colon were mobilized from their retroperitoneal attachments according to the principle of complete mesocolic excision (CME). Then, the proximal and distal intestines were transected with two 60-mm linear staplers at least 10 cm from the tumor (Fig. 2a, b). Overlapped delta-shaped anastomosis was performed intracorporeally using linear staplers in all the cases as described previously [10]. A side-to-side isoperistaltic anastomosis is fashioned using a 60-mm linear stapler (Fig. 2c), and the common enterotomy was closed using another 60-mm linear stapler (Fig. 2d). The specimen (Fig. 3a, b) was removed either from the anus, abdominal scar of previous surgery, or from a Pfannenstiel incision that was made 2 to 3 cm above the symphysis pubis at the border of the pubic hair.

For the control group, the origins of the ileocolic vessels were identified and then ligated at their origin point. Then the right colic vessels (if they existed), the sRCV, the RGEV, and the middle colic vessels were also isolated and ligated at their origins. Hypopyloric lymphadenectomy was also performed along with the original ligation of the RGEA. After mobilization of the relevant parts of the colon from their retroperitoneal attachments, the intestines were transected by two laparoscopic linear staplers at the terminal ileum approximately 15 cm from the ileocecal junction and transverse colon at least 10 cm distal to the tumor. The anastomosis and the removal of specimen were performed using the same approach as the ileocecal junction-preserved group.

### Follow-up

The first day after the surgery represented the beginning of the follow-up period. During hospitalization, the surgeon assessed the patient’s recovery state during daily rounds. Follow-up after discharge was performed by telephone that lasted for at least 2 weeks after the operation. Patients were routinely followed up at outpatient clinics every 3 months after the operation in the first 2 years and every 6 months thereafter. Recurrence of cancer and distant metastasis were monitored on the basis of physical and laboratory examinations including biomarkers (CEA and CA-199) at each visit, CT scans of the chest, abdomen, and pelvis at every half year and a complete colonoscopy every year.

### Statistical analysis

Statistical analysis was performed using the SPSS software, version 22.0 for Windows (SPSS Inc., Chicago, IL, USA). Distribution of the data was checked for normality using the Kolmogorov–Smirnov test. Non-parametric continuous variables are presented as median and interquartile range (IQR) and were compared using the Mann–Whitney *U* test. Normally distributed continuous variables were presented as the mean and standard deviations and were compared using the Student *t* test. Qualitative variables were given as the number and percentage and were compared with the *χ*^2^ test. *P* values of less than 0.05 were considered statistically significant.

## Results

### General data

As shown in Table 1, although the BMI of the control group was higher than the ileocecal junction-preserved group (*p* = 0.029), there were no significant differences in the patients’ demographics in relation to age, gender, ASA scores, previous abdominal operation history, and preoperative chemotherapy between the two groups (*p* > 0.05).

**Table 1:** Clinical characteristics of patients

	Ileocecal junction-preserved group (*n* = 23)	Control group (*n* = 34)	*P* value
Gender, *n* (%)			0.968
Male	15 (65.2)	22(64.7)	
Female	8 (34.8)	12(34.3)	
Age, year, median (IQR)	62.0 (56.0–71.0)	64.0 (59.5–71.3)	0.397
BMI, kg/m^2^, median (IQR)	26.0 (23.1–27.9)	23.7 (21.3–25.9)	0.029
ASA score, *n* (%)			1.000
1–2	22 (95.7)	33 (97.1)	
3–4	1 (4.3)	1 (2.9)	
Previous abdominal operation, *n* (%)			0.917
Yes	5 (21.7)	7 (20.6)	
No	18 (78.3)	27 (79.4)	
Preoperative neoadjuvant therapy, *n* (%)			
NACT	6 (26.1)	6 (17.6)	0.443
No	17 (73.9)	28 (82.4)	

### Surgical and pathological data

All patients in this study underwent complete laparoscopic surgery successfully. Surgical and postoperative data are presented in Table 2. The median operation time for the ileocecal junction-preserved group was 120.0 min, which was significantly shorter than 132.0 min for the control group (*p* = 0.048). The intraoperative blood loss (*p* = 0.655) was similar between the two groups. In majority of the cases, the specimen was removed from the Pfannenstiel incision and no intraoperative or anesthetic complications occurred.

**Table 2:** Surgical and pathological outcomes of patients

	Ileocecal junction-preserved group (*n* = 23)	Control group (*n* = 34)	*P* value
Operation time, min, median (IQR)	120.0 (110.0–123.0)	132.0 (110.0–155.0)	0.048
Intraoperative blood loss, ml, median (IQR)	20.0 (10.0–60.0)	20.0 (10.0–60.0)	0.655
Removal method of the specimen, *n* (%)			0.858
Natural orifice	1 (4.3)	2 (5.9)	
Abdominal scar	3 (13.0)	3 (8.8)	
Pfannenstiel incision	19 (82.6)	29 (85.3)	
The length of tumor, cm, median (IQR)	3.0 (3.0–4.0)	3.3(3.0–4.3)	0.664
Proximal resection margin, cm, median (IQR)	11.3 (10.0–13.8)	24.0 (23.0–24.6)	< 0.001
Distal resection margin, cm, median (IQR)	11.0 (10.0–12.0)	11.0 (10.0–13.3)	0.345
Number of lymph nodes retrieved, median (IQR)	27.0 (23.0–34.0)	32.5 (25.0–44.0)	0.083
Number of metastatic lymph nodes, median (IQR)	0 (0–1.0)	0 (0-1.0)	0.905
Rate of metastatic lymph nodes (%)	9 (39.1)	14 (41.2)	0.877
*p*TNM stage, *n* (%)			0.674
I	1 (4.3)	4 (11.8)	
II	13 (56.5)	16 (47.1)	
III	19 (39.1)	14 (41.2)	

There was no difference in the length of the tumor (*p* = 0.664) and distal resection margin (*p* = 0.345), although the proximal resection margin was shorter in the ileocecal junction-preserved group than in the control group (11.3 cm vs. 24.0 cm, *p* < 0.001). All patients received a D3 LN dissection, and there was no difference in the number of the harvested lymph nodes between two groups, although the median number was higher in the control group (27.0 vs. 32.5, *p* = 0.083). There was also no difference in the number of metastatic lymph nodes and rate of metastatic lymph nodes (*p* > 0.05). Tumor TNM stage was well balanced in both groups (*p* =0.674).

### Postoperative general recovery and complications

Table 3 shows that the time to ground activities (*p* = 0.742) and fluid diet intake (*p* = 0.213) were similar between the two groups, while the time to first flatus (*p* = 0.010) and the duration of postoperative hospitalization (*p* = 0.017) were significantly shorter in the ileocecal junction-preserved group than in the control group. We also found no significant differences between the groups in terms of the cost of hospitalization (*p* = 0.097).

**Table 3:** Postoperative general recovery and complications of patients

	Ileocecal junction-preserved group (*n* = 23)	Control group (*n* = 34)	*P* value
First ground activities, days, median (IQR)	1.0 (1.0–2.0)	1.0 (1.0–2.0)	0.742
First flatus passage, days, median (IQR)	3.0 (2.0–3.0)	3.0 (3.0–4.0)	0.010
Fluid diet intake, days, median (IQR)	1.0 (1.0–2.0)	1.0 (1.0–2.0)	0.213
Postoperative hospitalization, days, median (IQR)	6.0 (4.0–7.0)	6.0 (6.0–7.0)	0.017
Hospitalization cost, USD, median (IQR)	10306.3 (9819.4–10673.5)	9476.8 (8914.8–10378.9)	0.097
Postoperative diarrhea, *n* (%)	4 (17.4)	16 (47.1)	0.026
Clavien–Dindo complications, *n* (%)	4 (17.4)	7 (20.6)	1.000
I–II	4 (17.4)	6 (17.6)	
III–IV	0	1 (3.0)	
Reoperation, *n* (%)	0	0	-
Readmission, *n* (%)	0	0	-
Mortality, *n* (%)	0	0	-

The mean follow-up period between two groups was 22.2 months (range, 12–31 months; ileocecal junction-preserved group, 21.6 months; control group, 22.6 months). The occurrence of postoperative diarrhea was higher in the control group than in the ileocecal junction-preserved group (17.4% vs. 47.1%, *p* = 0.026). The other postoperative complications were comparable between the two groups with no significant differences according to the Clavien–Dindo classification (*p* = 0.1.00). The most common morbidities in the ileocecal junction-preserved group were wound infections in two patients (8.7%), followed by anastomosis bleeding in one patient (4.3%), and pneumonia in one patient (4.3%). In the control group, the most common morbidities were also wound infections in three patients (8.8%), followed by lymphorrhea in two patients (5.9%), abdominal infection in one patient (2.9%), and bowel obstruction in one patient (2.9%). All complications were resolved successfully.

### Postoperative bowel function recovery

Most poor continence occurred within 3 months after surgery. Table 4 shows that the defecation frequency was lower in the ileocecal junction-preserved group than in the control group on the 1st, 3rd, and 6th month (*p* < 0.05) and became comparable on the 12th month (*p* = 0.251). The number of patients who defecated at night or defecated four times or more a day was less in the ileocecal junction-preserved group than control group on the 1st month (*p* < 0.05) and became comparable on the 3rd, 6th, and 12th month (*p* > 0.05).

**Table 4:** Postoperative bowel function recovery of patients

	Ileocecal junction-preserved group (*n* = 23)	Control group (*n* = 34)	*P* value
First month after surgery			
Defecation frequency, *n*, median (IQR)	2.0 (1.0-3.0)	3.0 (3.0-4.0)	< 0.001
Defecation at night, *n* (%)	2 (8.7)	12 (35.3)	0.029
Defecation four times or more a day, *n* (%)	3 (13)	14 (42.4)	0.022
Third month after surgery			
Defecation frequency, *n*, median (IQR)	2.0 (1.0-3.0)	3.0 (2.0-3.0)	0.004
Defecation at night, *n* (%)	2 (8.7)	7 (20.6)	0.288
Defecation four times or more a day, *n* (%)	2 (8.7)	8 (23.5)	0.178
Sixth months after surgery			
Defecation frequency, *n*, median (IQR)	1.0 (1.0–2.0)	2.0 (1.0–2.0)	0.018
Defecation at night, *n* (%)	1 (4.3)	3 (8.8)	0.641
Defecation four times or more a day, *n* (%)	1 (4.3)	6 (17.6)	0.223
Twelfth month after surgery			
Defecation frequency, *n*, median (IQR)	1.0 (1.0-1.0)	1.0 (1.0-2.0)	0.251
Defecation at night, *n* (%)	0	1 (2.9)	1.000
Defecation four times or more a day, *n* (%)	0	2 (5.9)	0.510

## Discussion

The principle of CME has been adopted for many years with solid evidence and strong support by more and more surgeons as the optimal approach for colon cancer surgery [11–13]. It is believed that a more complete lymphadenectomy can increase survival rates so the frequency of CME with central vascular ligation (CVL) for right-sided colon cancer is on the rise. For right-side colon cancer, the resection of ileocecal junction, ascending colon, hepatic flexure, and transverse colon as well as lymphadenectomy along the middle colic, right colic, and ileocolic vessels is necessary [14]. The techniques above have become a standard surgical treatment in right-sided colon cancer.

However, the optimal surgical approach remains unclear for the treatment of transverse colon cancer, and it is often based on the surgeon’s preference whether to perform an extended colectomy or a transverse colectomy. In several retrospective studies for patients with transverse colon cancer, it was [15, 16] demonstrated that there were no differences between an extended colectomy and a transverse colectomy in terms of postoperative risk and oncological outcomes, and concluded that a segmental resection may be considered as an option for the treatment of localized tumors of the transverse colon. Theoretically, Toyota et al. [6] found that for 24 patients (45.2%) with lymph node metastasis who were identified among the 53 patients with right-transverse colon cancer, there was no lymph node metastasis at the root of the ileocolic artery. Moreover, a study investigating the extent of lymph node metastasis found that in 164 cases of right colon cancer with lymph node metastasis, the vast majority of positive nodes was located less than 10 cm from the lesion, regardless of the position of the cancer [17]. Some studies also found that the longitudinal spread was only observed in the N1 zone (within 5 cm) and in the N2 (within 10 cm) pericolic station [18]. For left-sided colon cancers, longitudinal spread greater than 10 cm beyond the tumor was not found, and the data for right-sided tumors is only 1 to 4% [19]. According to the Japanese guidelines [18], longitudinal metastatic lymph nodes are rarely found greater than 10 cm beyond the tumor; therefore, resection of a 10-cm segment of normal bowel both in the proximal and distal zones to the tumor is adequate. In contrast, for patients with metastases in epicolic and paracolic nodes greater than 10 cm from the tumor, a curative resection was not feasible. Therefore, it can be inferred that the segment of the normal bowel proximal to the tumor is much longer in conventional laparoscopic extended right hemicolectomy for right-transverse colon cancer. However, laparoscopic segmental colectomy for right-transverse colon cancer may be a difficult technique because the extracorporeal anastomosis (EA) needs long free intestines.

With improvements in surgical devices and technology, complete laparoscopic treatment for colon cancer with intracorporeal anastomosis (IA) has become widespread because of its advantages of being less invasive, having an earlier postoperative recovery time, and a lower complication rate when compared to laparoscopic-assisted surgery with EA [20, 21]. On the one hand, a recent meta-analysis including 3755 patients found that in IA, time to first flatus, time to defecation, time to liquid diet, hospital length of stay, postoperative infections, and overall complications were estimated to be lower [22]. On the other hand, Allaix et al. [8] found that the number of retrieved lymph nodes, the overall survival, and the disease-free survival at 3 years were not significantly different between IA and EA. The resection and anastomosis need not be performed extracorporeally for IA, and therefore a smaller portion of the intestine is required to be freed laparoscopically.

Based on the progress reported in theory and the techniques involved, we decided to perform complete laparoscopic extended hemicolectomy with preservation of the ileocecal junction to treat right-transverse colon cancer and explore its safety and feasibility. The technical difficulty and longer operation times may be challenging because of the new techniques involved and higher BMI in the ileocecal junction-preserved group. However, the median operation time for the ileocecal junction-preserved group was significantly shorter than the control group because the resection of the ileocecal junction and its related vessels were not needed. Therefore, we think the procedure with preservation of the ileocecal junction is less time-consuming, especially for experienced surgeons. We also found no difference between the amount of blood lost, which is one of most important evaluation parameters. In terms of postoperative recovery, a significantly shorter time of first flatus and an earlier recovery of defecating frequency were found in the ileocecal junction-preserved group. The main cause of the significantly different improvements in these parameters in postoperative recovery can be attributed to the preservation of the ileocecal valve. Previous studies found a higher hydro-electrolytic loss and a greater difficulty in adapting to the postoperative diet when the ileocecal valve was resected [23]. The alterations in microbiota caused by an increased resection of the intestine may also correlate with this finding.

Moreover, in the ileocecal junction-preserved group in our study, the pathological outcomes are identical when compared with the control group. Although the median lymph nodes yielded were fewer in the ileocecal junction-preserved group because of a lesser resection area of the intestine and mesentery, there was no difference between the two groups. The median achieved number was much greater than 12 nodes in the final surgical specimen count in both groups, meeting the demands of the TNM cancer staging system set by UICC (International Union Against Cancer) and American Joint Committee on Cancer [24]. There was no difference between two groups in the number of metastatic lymph nodes and rate of metastatic lymph nodes. Pathological diagnosis found negative resection margin in all the patients. Although the proximal resection margin was shorter in the ileocecal junction-preserved group, proximal and distal resection margins were more than 10 cm in the control group.

The limitations to our study were that it was a retrospective study and the present outcomes were from a single surgeon that represents a relatively small number of patients. However, the surgical procedures evaluated in this study were performed by an experienced surgeon with homogenous types of surgery and disease status of the patients. Prospective randomized controlled trials from multiple centers with larger sample sizes are now needed to confirm our results.

## Conclusion

Based on the results of our study, the complete extended laparoscopic hemicolectomy with preservation of the ileocecal junction proves to be a safe and feasible surgical procedure for right-transverse colon cancer. Potential benefits of preserving the ileocecal junction include simplified operating procedures, shorter operation times, similar pathological outcomes, and an earlier recovery of bowel function.

## Data Availability

The datasets generated and/or analyzed during the current study are not publicly available due to the data being a confidential patient data but are available from the corresponding author on reasonable request.

## Abbreviations

CRC: Colorectal cancer
BMI: Body mass index
ASA: American Society of Anesthesiologists
DFS: Disease-free survival
OS: Overall survival
SMV: Superior mesenteric vein
sRCV: Superior right colic vein
CME: Complete mesocolic excision
CVL: Central vascular ligation
EA: Extracorporeal anastomosis
IA: Intracorporeal anastomosis
UICC: International Union Against Cancer
